# Record of ^3^H and ^36^Cl from the Fukushima nuclear accident recovered from soil water in the unsaturated zone at Koriyama

**DOI:** 10.1038/s41598-023-46853-y

**Published:** 2023-11-11

**Authors:** Tomoko Ohta, L. Keith Fifield, László Palcsu, Stephen G. Tims, Stefan Pavetich, Yasunori Mahara

**Affiliations:** 1 Nagaoka University of Technology, Nagaoka, Japan; 2 The University of Tokyo, Kashiwa, Japan; 3 Australian National University, Canberra, Australia; 4 HUN-REN Institute for Nuclear Research, Debrecen, Hungary; 5 Kyoto University, Kyoto, Japan

**Keywords:** Environmental sciences, Hydrology

## Abstract

The opportunity to measure the concentrations of ^3^H and ^36^Cl released by the Fukushima nuclear accident in 2011 directly in rain was lost in the early stage of the accident. We have, however, been able to reconstruct the deposition record of atmospheric ^3^H and ^36^Cl following the accident using a bore hole that was drilled in 2014 at Koriyama at a distance of 60 km from the accident. The contributions of ^3^H and ^36^Cl from the accident are 1.4 × 10^13^ and 2.0 × 10^12^ atoms m^−2^ respectively at this site. Very high concentrations of both ^3^H (46 Bq L^−1^) and ^36^Cl (3.36 × 10^11^ atoms L^−1^) were found in the unsaturated soil at depths between 300 and 350 cm. From these, conservative estimates for the ^3^H and ^36^Cl concentrations in the precipitation in the ~ 6 weeks following the accident were 607 Bq L^−1^ and 4.74 × 10^10^ atoms L^−1^, respectively. A second hole drilled in 2016 showed that ^3^H concentrations in the unsaturated soil and shallow groundwater had returned to close to natural levels, although the ^36^Cl concentrations were still significantly elevated above natural levels.

## Introduction

**Figure 1:**
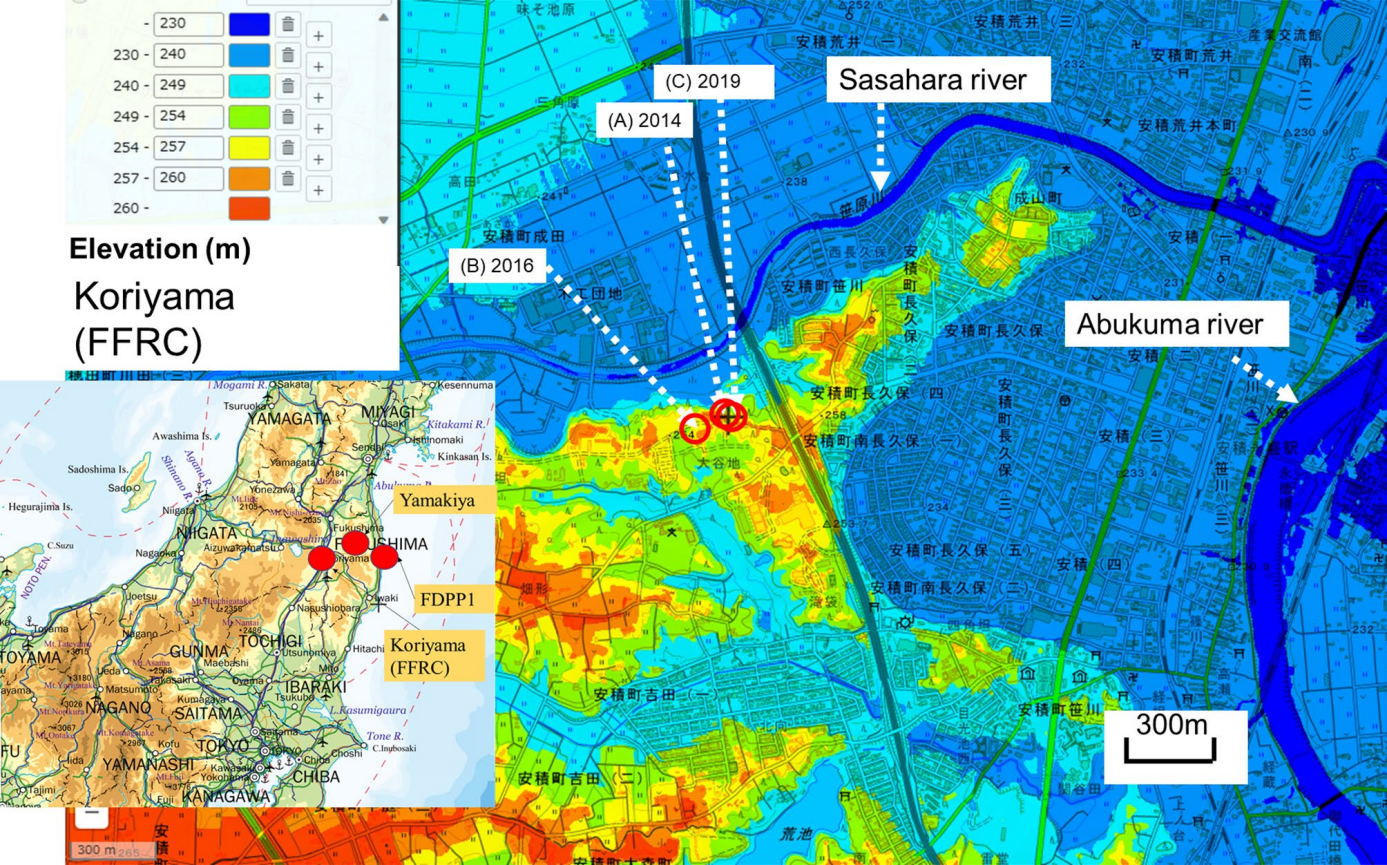
Locations of the soil cores and groundwater monitoring. FFRC: Fukushima Forestry Research Center. Core samples were collected in Koriyama (**A**), (**B**), Yamakiya. The variation in groundwater table was measured at site (**C**), as shown in Supplementary Fig. S4. The edited map is cited from the original map of (https://www.gsi.go.jp/tizu-kutyu.html). The map of the copyright holder is the Geospatial Information Authority of Japan.

Several studies in the past 50 years have exploited the bomb-pulse tritium released in nuclear weapon tests in unsaturated soil water^1,2^ and shallow groundwater^3–6^ across the globe as a hydro-tracer. A significant pulse of tritium was also released from the damaged reactors in the Fukushima nuclear accident, which was caused by a large earthquake and the resulting tsunami on March 11th, 2011. Rather than use this as a hydrological marker, however, the study reported here aims to reconstruct the amount of tritium deposited around the epicenter of the accident. To this end, a continuous depositional record of tritium was obtained from the soil water in a 6 m long drill-core through the unsaturated soil zone. It was collected in September 2014, 3.6 years after the accident, at the site of Koriyama (in the campus of the Fukushima Forestry Research Center 37.3576N, 140.3500E, Fig. 1), which is 60 km to the west-southwest of the Fukushima nuclear power plant 1 (FNPP1). The same site was resampled two years later in December 2016, and data were also collected in November 2016 at a site of Yamakiya (37.5920N, 140.7087E, Fig. 1), which is one of the areas in Fukushima prefecture that was heavily contaminated by the radioactive fallout.

The opportunity for direct measurement of tritium concentrations in precipitation over the extended area surrounding the plant in the aftermath of the accident was lost due to the human and mechanical disruptions caused by the earthquake. Some indirect data have been reported from the sap in herbaceous plants and evergreen tree leaves^7^ 50 km to the southwest (4 ~ 10 Bq L^−1^), from puddle water collected from 1.5 km from the FNPP1 (184 ± 2 Bq L^−1^)^8^, and from the mathematically estimated tritium activity in the air moisture^9^ by using the transfer factor (1 × 10^4^) and the measured tritium concentrations^9,10^ in rainwater collected at Tsukuba (170 km from the epicenter), Kashiwa (200 km), Tokyo (220 km) and Yokosuka (300 km) in late March of 2011. However, the lack of a direct measure of the tritium concentration released in the early stage of the nuclear accident introduces significant uncertainty into any future estimation of the internal radiation dose to humans caused by the accident.

Releases of the long-lived radioisotope ^36^Cl (T _½_ = 301,000 years) from the accident were also expected. The ^36^Cl/Cl ratios in surface soils at distances of 3–22 km from the power plant were reported 40 days after the accident^11^. Ratios between 1000 and 26,000 × 10^−15^ were observed, with the highest ratios to the W and SW of the plant, i.e. towards Koriyama. These ratios are much higher than natural ratios, which are expected to be ≤ 300 × 10^−15^ (Tosaki^12^). Only the top 5 cm of soil was sampled, however, and some of the deposited ^36^Cl may have been lost due to downward transport by the infiltrating soil water during the time that elapsed between the accident and the sampling. In contrast, and provided that the ^36^Cl and ^3^H were still within the unsaturated soil zone, the 6 m long soil core drilled in 2014 should preserve a complete record of post-accident deposition at the Koriyama site.

## Results and discussion

### Total inventories of ^3^H and ^36^Cl released by the accident

**Figure 2:**
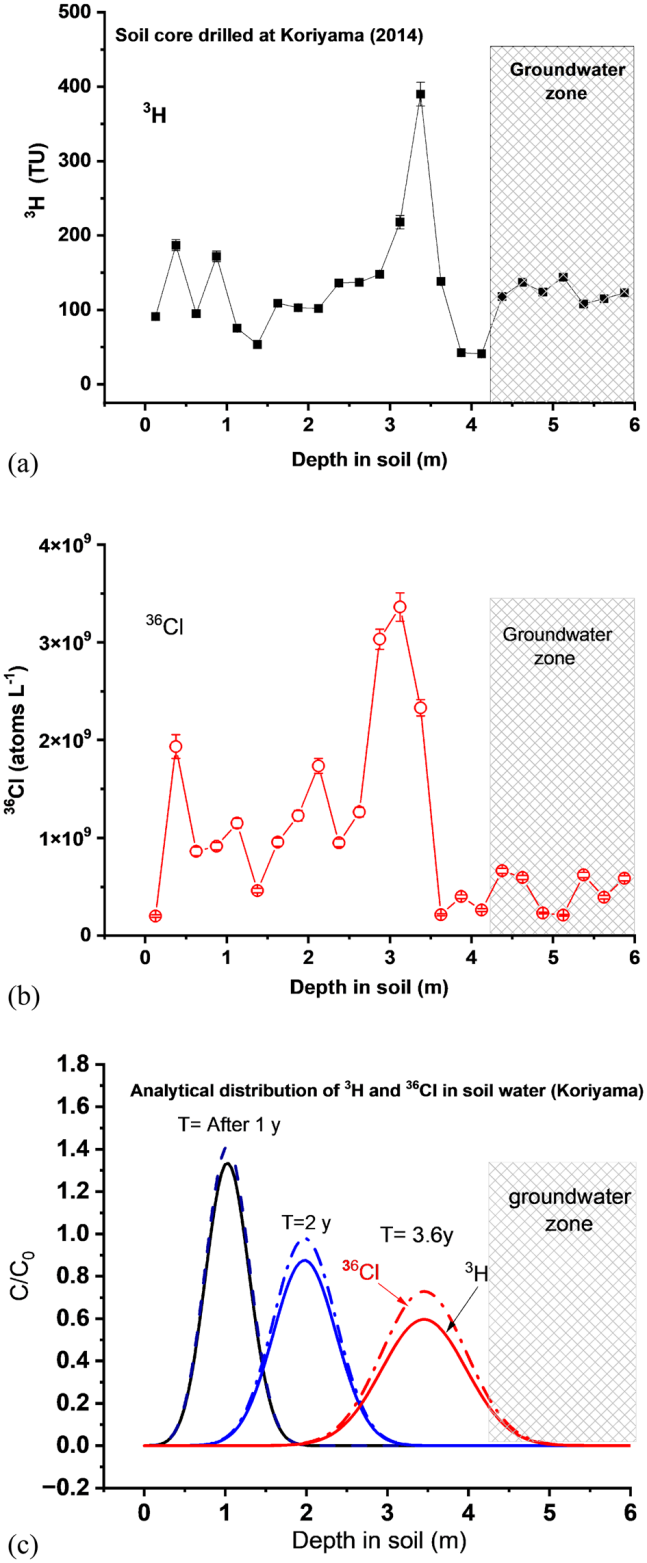
Correlation between the one-dimensional migration analyses in the unsaturated soil layer (0–6 m) and the vertical distribution of the ^3^H and ^36^Cl radionuclide released during the Fukushima nuclear accident, March 11th, 2011. (**a**) The distribution of the measured ^3^H (atoms L^−1^) concentration in the unsaturated soil (0.05–4.25 m) and saturated soil zones (4.25–6 m) on Sep. 24th, 2014, 3.6 years after the accident. (**b**) The distribution of the measured ^36^Cl concentration (atoms L^−1^) in the unsaturated soil (0.05–4.25 m) and saturated soil zones (4.25–6 m) on Sep. 24th, 2014. (**c**) Distributions of the hydro tracers (^3^H and ^36^Cl) transported by the rainwater that precipitated on March 15^th^, 2011, were estimated by one-dimensional analytical solutions via the dispersion (D = 0.039 m^2^ year^−1^) and vertical infiltration rate of soil water (v = 0.95 m year^−1^) in the unsaturated soil layer at Koriyama in Fukushima. Black solid (^3^H) and dash (^36^Cl) lines: 1 year later, blue line: 2 years later, red line: 3.6 years later. Decay of ^3^H has been included.

Concentrations of ^3^H and ^36^Cl together with detailed soil information (density, porosity, water content and saturation rate, and stable chloride concentration (mg kg^−1^)) were measured for each 25 cm interval within the undisturbed 6 m long soil core (Table 1 and Supplementary Table S1). Tritium in most of the samples was measured by beta-counting using a liquid-scintillation counter (LSC), while 6 samples were measured by the ^3^He in-growth method (see “Methods”). The ^36^Cl/Cl ratios were measured by accelerator mass spectrometry (see “Methods”). The results are shown in Fig. 2 and Table 1. Both the ^3^H and ^36^Cl concentrations show prominent peaks at a depth of ~ 3.4 m, about 1 m above the water table, although there is some indication that the ^36^Cl may lag the ^3^H slightly. This lag may be due to some of the ^36^Cl attaching to humic substances in the soil (ref. Sheppard^13^). The water table fluctuates between 4.25 and 4.75 m (see “Methods”). These findings are consistent with estimations using a one-dimensional advective diffusion transport model^14,15^ of the migration of a pulse of both ^3^H and ^36^Cl that fell out on the surface in rainfall in the immediate aftermath of the accident (Fig. 2 and “Methods”). These highest values, along with the elevated values throughout the profile, are dramatically higher than the natural concentrations, and are almost certainly attributable to releases following the accident.

**Table 1:** ^3^H and ^36^Cl concentration and magnitude of depositional flux of ^3^H and ^36^Cl at Koriyama (37.3576N, 140.3500E) and Yamakiya (37.5920N, 140.7087E) in Fukushima Pref. in Japan.

Koriyama 2014 depth (cm)		Sample ID	Infiltrated soil water flux (g cm^−2^)	^3^H (TU)	1σ	^3^H concentration (Bq L^−1^)	^3^H concentration (atoms L^−1^)	^3^H flux (atoms m^−2^) (1 Bq = 5.61E8 atoms)	(^36^Cl/Cl)_sample_ × 10^−15^	1σ	Cl in soil water (mg L^−1^)	^36^Cl (atoms L^−1^) in soil water	^36^Cl Flux (atoms m^−2^) (1 Bq = 1.37E13 atoms)
− 5*^1^	− 25	KRFC1-1	7.63	91.0	3.13	1.07E+01	6.02E+09	4.60E+11	528	33	22.44	2.01E+08	1.53E+10
− 25	− 50	KRFC1-2*	9.66	187	7.5	2.21E+01	1.24E+10	1.20E+12	3955	249	28.80	1.93E+09	1.87E+11
− 50	− 75	KRFC1-3*	8.93	95.2	3.8	1.12E+01	6.30E+09	5.63E+11	1504	77	33.82	8.63E+08	7.71E+10
− 75	− 100	KRFC1-4*	9.12	172	7	2.03E+01	1.14E+10	1.04E+12	2105	86	25.64	9.16E+08	8.35E+10
− 100	− 125	KRFC1-5*	10.60	75.5	3	8.91E+00	5.00E+09	5.30E+11	2722	120	24.95	1.15E+09	1.22E+11
− 125	− 150	KRFC1-6	10.92	53.7	0.5	6.33E+00	3.55E+09	3.88E+11	1046	55	26.02	4.61E+08	5.04E+10
− 150	− 175	KRFC1-7	12.76	109	1	1.29E+01	7.24E+09	9.24E+11	3378	148	16.72	9.58E+08	1.22E+11
− 175	− 200	KRFC1-8	12.42	103	1	1.21E+01	6.81E+09	8.45E+11	2897	125	25.02	1.23E+09	1.53E+11
− 200	− 225	KRFC1-9	12.07	102	1	1.20E+01	6.76E+09	8.15E+11	4870	211	21.00	1.74E+09	2.09E+11
− 225	− 250	KRFC1-10	11.86	136	1	1.60E+01	9.00E+09	1.07E+12	2688	113	20.84	9.50E+08	1.13E+11
− 250	− 275	KRFC1-11	11.92	137	1	1.62E+01	9.07E+09	1.08E+12	4894	193	15.25	1.27E+09	1.51E+11
− 275	− 300	KRFC1-12	8.47	148	1	1.75E+01	9.81E+09	8.31E+11	9152	311	19.53	3.03E+09	2.57E+11
− 300	− 325	KRFC1-13*	6.73	218	9	2.57E+01	1.44E+10	9.70E+11	12,700	550	15.61	3.36E+09	2.26E+11
− 325	− 350	KRFC1-14*	5.75	390	16	4.60E+01	2.58E+10	1.48E+12	9602	342	14.30	2.33E+09	1.34E+11
− 350	− 375	KRFC1-15	11.64	138	1	1.63E+01	9.13E+09	1.06E+12	1476	74	8.56	2.14E+08	2.49E+10
− 375	− 400	KRFC1-16	10.74	42.6	0.4	5.03E+00	2.82E+09	3.03E+11	2280	104	10.35	4.00E+08	4.30E+10
− 400	− 425	KRFC1-17	9.69	40.9	0.4	4.83E+00	2.71E+09	2.62E+11	1111	59	13.95	2.63E+08	2.55E+10
− 425	− 450	KRFC1-18	9.96	118	1	1.39E+01	7.79E+09	7.76E+11	4410	173	8.88	6.65E+08	6.62E+10
− 450	− 475	KRFC1-19	16.82	137	1	1.62E+01	9.09E+09	1.53E+12	9962	342	3.53	5.96E+08	1.00E+11
− 475	− 500	KRFC1-20	15.48	124	1	1.46E+01	8.19E+09	1.27E+12	4187	162	3.26	2.32E+08	3.59E+10
− 500	− 525	KRFC1-21	18.93	144	1	1.70E+01	9.54E+09	1.81E+12	4914	220	2.51	2.09E+08	3.96E+10
− 525	− 550	KRFC1-22	18.11	108	1	1.27E+01	7.13E+09	1.29E+12	4319	187	8.49	6.22E+08	1.13E+11
− 550	− 575	KRFC1-23	15.40	115	1	1.35E+01	7.60E+09	1.17E+12	3651	172	6.36	3.94E+08	6.07E+10
− 575	− 600	KRFC1-24	13.12	123	1	1.45E+01	8.12E+09	1.07E+12	5002	232	6.92	5.87E+08	7.70E+10

Koriyama 2016 depth (cm)		Sample ID	Infiltrated soil water flux (g cm^−2^)	^3^H (TU)	1σ	^3^H concentration (Bq L^−1^)	^3^H concentration (atoms L^−1^)	^3^H flux (atoms m^−2^) (1 Bq = 5.61E8 atoms)	(^36^Cl/Cl)_sample_ × 10^−15^	1σ	Cl in soil water (mg L^−1^)	^36^Cl (atoms L^−1^) in soil water	^36^Cl flux (atoms m^−2^) (1 Bq = 1.37E13 atoms)
− 35	− 50	KFRC35-50	5.15	1.71	2.27	2.02E−01	1.13E+08	5.83E+09	58	12	29.5	2.92E+07	1.51E+09
− 135	− 150	KFRC135-150	8.23	6.31	2.56	7.45E−01	4.18E+08	3.44E+10	436	39	8.9	6.57E+07	5.41E+09
− 273	− 300	KY273-300	5.70	7.57	2.57	8.93E−01	5.01E+08	2.86E+10	166	28	11.9	3.34E+07	1.90E+09
− 392	− 400	KY392-400	2.12	3.87	2.48	4.57E−01	2.56E+08	5.44E+09	199	24	59.3	2.00E+08	4.25E+09
− 480	− 500	KY480-500	5.53	0.85	2.4	1.00E−01	5.63E+07	3.11E+09	173	28	21.7	6.36E+07	3.52E+09
− 530	− 547	KY530-550	5.69	5.57	2.66	6.57E−01	3.69E+08	2.10E+10	153	18	14.4	3.72E+07	2.12E+09
− 581	− 596.5	KY581-600	5.56	7.26	3.06	8.57E−01	4.81E+08	2.67E+10	227	26	8.0	3.08E+07	1.71E+09
− 635	− 650	KFRC635-650	5.32	1.30	2.56	1.53E−01	8.61E+07	4.57E+09	176	25	12.4	3.71E+07	1.97E+09
− 732	− 752	KFRC732-752	9.79	1.23	2.26	1.45E−01	8.14E+07	7.97E+09	284	38	6.6	3.16E+07	3.10E+09

Yamakiya 2016 depth (cm)		Sample ID	Infiltrated soil water flux (g cm^−2^)	^3^H (TU)	1σ	^3^H concentration (Bq L^−1^)	^3^H concentration (atoms L^−1^)	^3^H flux (atoms m^−2^) (1 Bq = 5.61E8 atoms)	(^36^Cl/Cl)_sample_ × 10^−15^	1σ	Cl in soil water (mg L^−1^)	^36^Cl (atoms L^−1^) in soil water	^36^Cl flux (atoms m^−2^) (1 Bq = 1.37E13 atoms)
− 80	− 100	YM-2016-4	7.11	3.83	2.60	0.452	2.54E+08	1.80E+10	705	58	9.58	1.15E+08	8.15E+09
− 177	− 194	YM-2016-8	5.91	4.95	2.64	0.584	3.28E+08	1.94E+10	214	38	8.38	3.04E+07	1.80E+09
− 278	− 295	YM-2016-12	3.42	1.11	2.54	0.131	7.35E+07	2.52E+09	283	55	10.84	5.21E+07	1.78E+09
− 382	− 400	YM-2016-16	4.08	4.18	2.49	0.493	2.77E+08	1.13E+10	146	37	12.68	3.14E+07	1.28E+09
− 450	− 465	YM-2016-19	2.78	3.64	2.21	0.430	2.41E+08	6.70E+09	339	79	9.90	5.70E+07	1.58E+09
− 500	− 514.5	YM-2016-21	3.03	7.91	2.79	0.934	5.24E+08	1.59E+10	257	37	11.75	5.13E+07	1.55E+09
− 550	− 564.5	YM-2016-23	3.50	6.46	2.85	0.762	4.28E+08	1.50E+10	289	37	11.40	5.59E+07	1.96E+09
− 600	− 612	YM-2016-25	2.29	4.39	2.24	0.518	2.90E+08	6.65E+09	220	36	11.44	4.27E+07	9.77E+08
− 650	− 664	YM-2016-27	3.11	0.50	2.12	0.059	3.28E+07	1.02E+09	205	36	11.77	4.09E+07	1.27E+09
− 783	− 800	YM-2016-32	3.93	6.60	2.69	0.779	4.37E+08	1.72E+10	354	47	13.75	8.25E+07	3.25E+09

Koriyama 2020 depth (cm)		Sample ID	Infiltrated soil water flux (g cm^−2^)	^3^H (TU)	1σ	^3^H concentration (Bq L^−1^)	^3^H concentration (atoms L^−1^)	^3^H flux (atoms m^−2^) (1 Bq = 5.61E8 atoms)	(^36^Cl/Cl)_sample_ × 10^−15^	1σ	Cl in soil water (mg L^−1^)	^36^Cl (atoms L^−1^) in soil water	^36^Cl flux (atoms m^−2^) (1 Bq = 1.37E13 atoms)
− 680		Groundwater	–	2.4	1.1	0.283	1.59E+08	–	325	9	9.0	4.96E+07	–

Koriyama 2021 depth (cm)		Sample ID	Infiltrated soil water flux (g cm^−2^)	^3^H (TU)	1σ	^3^H concentration (Bq L^−1^)	^3^H concentration (atoms L^−1^)	^3^H flux (atoms m^−2^) (1 Bq = 5.61E8 atoms)	(^36^Cl/Cl)_sample_ × 10^−15^	1σ	Cl in soil water (mg L^−1^)	^36^Cl (atoms L^−1^) in soil water	^36^Cl flux (atoms m^−2^) (1 Bq = 1.37E13 atoms)
− 651		Groundwater	–	3.2	1.1	0.378	2.12E+08	–	313	9	10.2	5.42E+07	–

*Tritium concentration in soil water was measured by the ingrowth method, *1: surface soil between 0 and 5 cm was removed due to heavy contamination of radiocesium.

**Depositional Flux: inventory (Bq m^−2^).

***^3^H flux and ^36^Cl flux: inventories of ^3^H and ^36^Cl.

From the data given in Table 1 for the 2014 core, the total inventories of ^3^H and ^36^Cl released by the accident and rained out at Koriyama can be estimated. Between 0.05 m and the water table at 4.25 m there are (1.38 ± 0.01) × 10^13^ and (1.99 ± 0.03) × 10^12^ atoms m^−2^ of ^3^H and ^36^Cl respectively. Natural cosmic-ray produced ^3^H and ^36^Cl contribute only a small fraction, 2.1 × 10^12^ and 4.5 × 10^9^ atoms m^−2^ respectively, where the natural fallouts of ^3^H and ^36^Cl in Japan are taken to be 6 tritium units (TU where 1 TU = 6.6 × 10^7^ atoms L^−1^ = 0.118 Bq L^−1^) (Mahara and Igarashi^16^) and 32 atoms m^−2^ s^−1^ (Tosaki et al*.*^12^) respectively. Hence the contributions of ^3^H and ^36^Cl at the Koriyama site from the Fukushima accident at the time of sampling are 1.17 × 10^13^ and 1.99 × 10^12^ atoms m^−2^ respectively. These can be converted into an estimate of the concentrations in rainwater in the immediate aftermath of the accident as follows. First, the tritium inventory must be decay corrected from the time of collection (24 September 2014) to the time activity of the accident (11 March 2011), resulting in 1.43 × 10^13^ atoms m^−2^. Then, based on the results of Matsumoto et al*.*^9^, who measured a time series of tritium concentrations in rainfall at a number of sites around Japan after the accident, and found that the concentrations had returned to natural levels by late April 2011 in all cases, we have assumed that, at the Koriyama site, essentially all of the ^3^H and ^36^Cl fell out in the 42 mm of rain that fell between March 12^th^ and April 20^th^, 2011. The corresponding average ^3^H and ^36^Cl concentrations in this rainwater were then 607 (Bq L^−1^) (3.41 × 10^11^ atoms L^−1^, 5100 TU) and 3.46 mBq L^−1^ (4.74 × 10^10^ atoms L^−1^), respectively. We emphasize that these values are conservative estimates. It is likely that most of the activity fell out before the end of March, in which case the average concentrations in the 16 mm of rain that fell between 11 and 31 March would be significantly higher at 1594 Bq L^−1^ and 9.1 mBq L^−1^ for ^3^H and ^36^Cl respectively. Even these higher values, however, are an order of magnitude below the WHO guidelines for permissible levels in drinking water of 1 × 10^4^ Bq L^−1^ for ^3^H and 100 Bq L^−1^ for ^36^Cl (WHO^17^). Hence, the human health impacts of the ^3^H and ^36^Cl releases from the accident would have been negligible in the vicinity of Koriyama.

It is also evident from the 2014 data presented in Table 1 that the groundwater below the water table already contained elevated levels of ^3^H and ^36^Cl that would not have been expected on the basis of the modelled profiles shown in Fig. 2c. These elevated levels are attributed to preferential flow paths arising from mining activity to extract brown coal during World War II. See Supplementary Information S1 and Supplementary Fig. S3.

### Environmental recovery of the nuclides left in the underground environment

**Figure 3:**
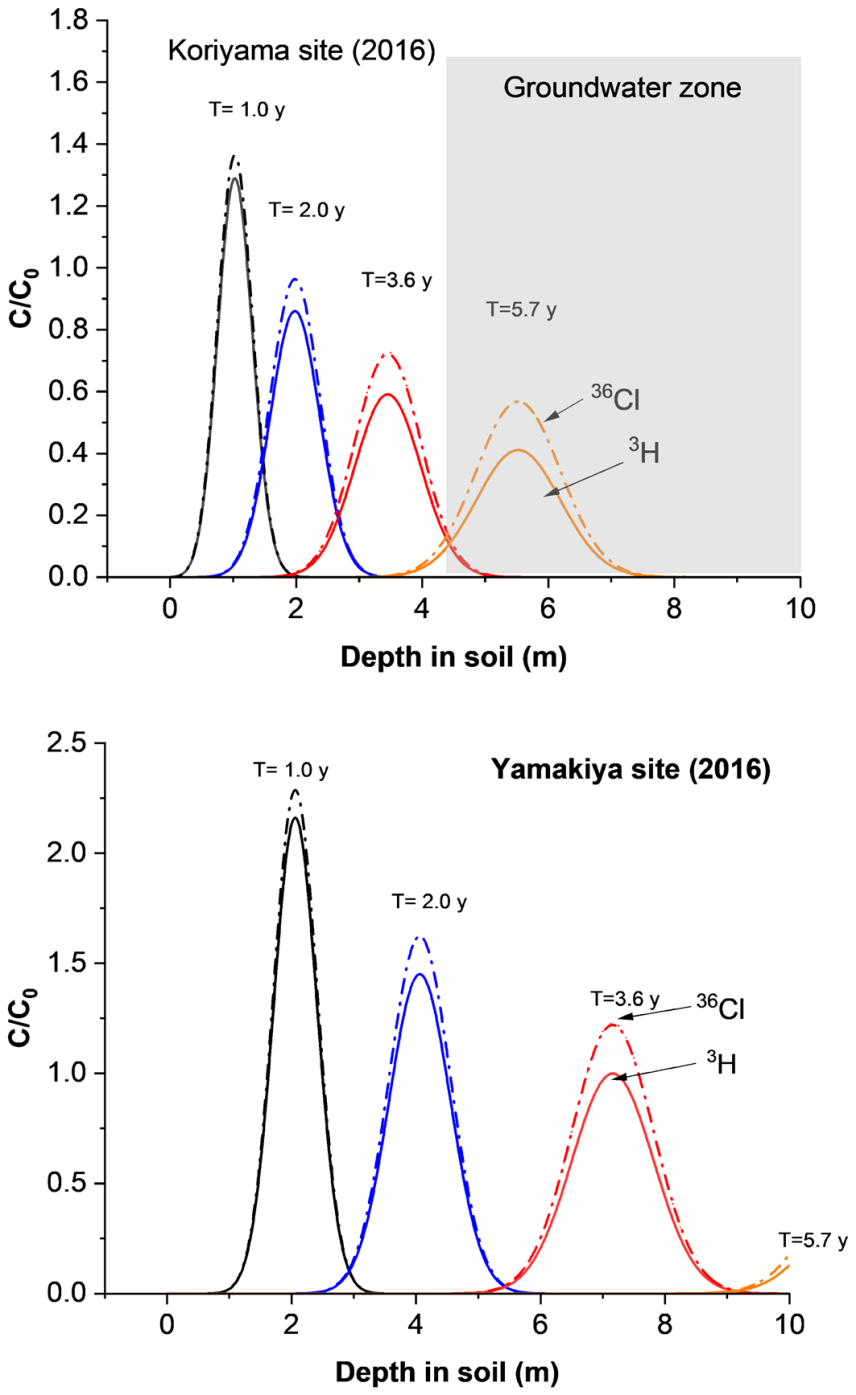
Distributions of the hydro tracers (^3^H and ^36^Cl) transported by the rainwater that precipitated on March 15th, 2011, were estimated by one-dimensional analytical solutions. (**a**) Koriyama in Fukushima Prefecture. Black solid (^3^H) and dash (^36^Cl) lines: 1 year later, blue line: 2 years later, red line: 3.6 years later, yellow dashed line: 5.7 years later. One-dimensional analytical solutions were estimated via the dispersion (D = 0.039 m^2^ year^−1^) and vertical infiltration rate of soil water (v = 0.95 m year^−1^) in the unsaturated soil layer. (**b**) Yamakiya in Fukushima Prefecture. Black solid (^3^H) and dash (^36^Cl) lines: 1 year later, blue line: 2 years later, red line: 3.6 years later, yellow dashed line: 5.7 years later. One-dimensional analytical solutions were estimated via the dispersion (D = 0.060 m^2^ year^−1^) and vertical infiltration rate of soil water (v = 2 m year^−1^) in the unsaturated soil layer. Yamakiya belongs to granite area which has flow-path fractures and the vertical infiltration rate would be ≥ 2 m year^−1^. The water table is below 10 m.

**Figure 4:**
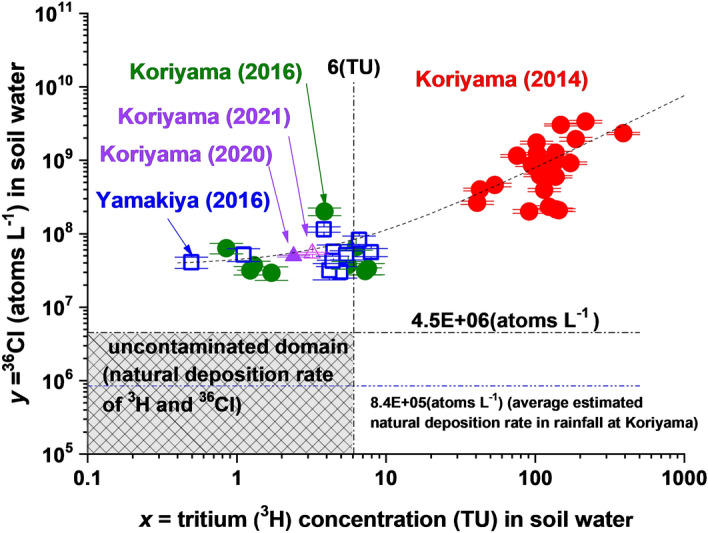
The distributions of the ^3^H (TU) and ^36^Cl (atoms L^−1^) concentrations in unsaturated soil water (collected in 2014 at Koriyama and in 2016 at Koriyama and Yamakiya) and shallow groundwater (collected at Koriyama in 2020 and 2021 at a depth of 6.8 m and 6.5 m below the surface) relative to the zone of natural ^3^H (< 6 TU) and ^36^Cl (< 4.6 × 10^6^ atoms L^−1^) deposition measured in 2014 and 2016 in Fukushima. The light gray area indicates the possible range of natural fallout of ^3^H and ^36^Cl. The black dashed line shows the maximum ^36^Cl concentration deduced from the maximum deposition flux in the spring peak observed at Tsukuba^12^ and annual precipitation of Koriyama. The blue dashed line shows the average natural concentration of ^36^Cl estimated from the average annual deposition flux observed at Tsukuba^12^ and annual precipitation of Koriyama.

In order to investigate the subsequent passage of these radionuclides down the soil column, two additional continuous soil cores were collected ~ 2 years later at two different locations in the Fukushima prefecture. One, with a length of 10 m was again taken at Koriyama, while the other was drilled at Yamakiya to a depth of 10 m. In addition, groundwater from a depth of 6.8 m and 6.5 m, i.e. below the water table, was sampled in 2020 and 2021. Concentrations of ^3^H and ^36^Cl in selected sections of these two cores and in the later groundwater samples are also shown in Tables 1 and S1. As expected from the modelled profile shown in Fig. 3a, the excess ^3^H and ^36^Cl from the accident have been largely washed out from the soil column by late 2016, 5.7 years after the accident. This is depicted graphically in Fig. 4, where ^36^Cl and ^3^H concentrations are plotted against each other. The shaded region indicates the natural domain of cosmic ray produced ^3^H and ^36^Cl, which was defined as follows. For tritium, the average concentration in rainfall prior to the nuclear testing era was estimated by Mahara and Igarishi^16^ to be ~ 6 TU, based on the excess tritiogenic ^3^He concentration in groundwater near the boundary between shallow and deep groundwater at a site in northern Japan. It is also the conservative background value adopted by Matsumoto et al*.*^9^ based on measurements in rainfall over several years. Consequently, we adopt a conservative upper limit of 6 TU in Fig. 4. For ^36^Cl, the natural range can be estimated conservatively from the data of Tosaki et al*.*^12^, who measured concentrations between 2 × 10^5^ and 3.8 × 10^6^ atoms L^−1^ (deposition rate between 8.2 and 173.2 atoms m^−2^ s^−1^) in rainfall sampled monthly over a period of 5 years at Tsukuba. A conservative upper limit for the natural concentration of ^36^Cl in soil water and groundwater at Koriyama is then 4.6 × 10^6^ atoms L^−1^, which is based on the deposition rate at the spring peak at Tsukuba and precipitation at Koriyama. The long-term average concentration of 8.44 × 10^5^ atoms L^−1^, based on the average ^36^Cl deposition rate observed at Tsukuba (ref. Tosaki) and annual precipitation at Koriyama, is also indicated in Fig. 4. It is evident that the 2014 data for both ^3^H and ^36^Cl lie well outside the domain of natural fallout. By 2016, the data have moved much closer to the natural domain, but still fall significantly above it. Although many of the 2016 tritium values fall below the 6 TU limit, several values are above 6 TU, and the average tritium concentration is rather high at 8.3 TU. All of the ^36^Cl concentrations measured in 2016, however, fall well above the natural domain. Further, these elevated levels persist through into the groundwater samples collected in 2020 and 2021 at Koriyama, whereas tritium is at natural levels in these samples. These observations indicate that, while tritium had largely returned to natural levels by 2016, there was still an excess of ^36^Cl in the environment even in 2021, perhaps due to continued release from organic litter near the soil surface.

## Conclusion

In summary, accurate estimates of the total inventories of ^3^H and ^36^Cl that fell out at Koriyama as a consequence of the Fukushima accident have been recovered from the soil water in the unsaturated zone from a core drilled in 2014. From these, the concentration of the two radioisotopes in rainwater at the time have been estimated to be 607 Bq L^−1^ and 3.46 mBq L^−1^ for ^3^H and ^36^Cl respectively under the assumption that the bulk of the activity fell out in the ~ 6 weeks following the accident. By 2016, most of the tritium had gone from the unsaturated zone, although ^36^Cl concentrations were still significantly elevated above natural (cosmic-ray produced) levels.

## Methods

### Collection of soil core to determine the concentrations of ^36^Cl and ^3^H and the seasonal variations in δD and δ^18^O in soil water

Soil cores were collected in Koriyama, Fukushima prefecture, on September 24, 2014, and December 1, 2016, and from Yamakiya, Fukushima, on November 29, 2016 (Fig. 1). To protect the original tritium concentration in soil water in the cores, they were drilled without drilling water. After collection, the 2014 Koriyama core was sectioned into 25 cm lengths, while the lengths of the sections of the 2016 cores varied between 8 and 27 cm (see Table 1). After sectioning, the samples were immediately wrapped in plastic and frozen. The frozen samples were then shipped to the laboratory. Core profiles are shown in Supplementary Fig. S2.

### Geological settings of the three bore sites at Koriyama

Two soil cores were collected at the Koriyama site in 2014 and 2016 to depths of 6 m and 10 m respectively, and another hole was drilled to 8 m in 2019 in order to observe fluctuations in the water table. The water level in this latter hole was monitored every minute from 29 January 2019 to 13 December 2021. All 3 cores were drilled on a small tableland which varies in height from 240 to 259 m above sea-level and is approximately 30 m above the Sasahara River (Fig. 1, Supplementary Fig. S1).

This highland area is rich in brown coal and was actively mined during World War II by the primitive method of gophering. These workings were roughly filled in and abandoned after the war, and no record of their locations has survived. Some small caves and depressions have recently been found within the Koriyama forestry research campus^18,19^ which may be evidence of these activities. It is likely that there were such workings near our drilling sites, since a layer of brown coal (Ov: Organic viscous in Supplementary Fig. S2) was found at a depth of 4.25 m and 7 m in the cores drilled in 2014 and 2016, respectively (Although not exactly the same soil character, soil character of the 2014 core is similar to the 2016 core). These provide preferential flow paths to the water table^20^, which may explain why the ^3^H and ^36^Cl levels below the water table are already elevated in the 2014 core (Supplementary Fig. S3). Although secondary peaks in the ^3^H concentration within the first 1-m interval below the surface layer of the 2014 soil core, the peak may be caused by (1) rich organic matter in the shallow forestry soil and litter or (2) the diffusion of tritium, or isotopic exchanges with tritium, occurred on the swelling clayey soil particles. Future studies will need to check the origin of the unknown tritium peaks in the shallow soil zone.

### Annual variation in the groundwater table in the drilling hole

The groundwater table in the observation well drilled in 2019 was measured by a water table gauge (S&DL mini Model 4800, OYO) combined with a barometer for atmospheric pressure compensation. The variation in the groundwater table in the borehole from 2019 to 2021 is shown in Supplementary Fig. S4. At this site, the annual variability of the groundwater table ranged between a low in the winter and a high in summer to autumn. The depth of the groundwater table in the well (Supplementary Fig. S1C) was found to vary over the course of a year by approximately 50 cm from 6.4 to 6.9 m. Therefore, the depth of the groundwater table of 2014 boring in Supplementary Fig. S1A was estimated to vary over the course of a year by approximately 50 cm from 4.25 to 4.75 m.

This well (Supplementary Fig. S1C) also provided the groundwater samples from 6.8 m and 6.5 m depth in October 2020 and December 2021.

### Extraction of Cl isotopes from the soil core and stable Cl measurement by ion chromatography

After separation of the soil water from the core by distillation for the ^3^H analysis, the Cl that originated from the soil water remained in the dried soil. The dried samples were homogenized by mixing with a mortar and pestle. Chloride was extracted from a total of 200 g of dry soil by placing 10 g of the homogenized soil in each of 20 centrifuge tubes and adding 20 ml of ultrapure water to each. The tubes were then shaken for 1 day to extract the chloride into solution. The mixture was centrifuged at 3500 rpm to separate the solution (C_1_) from the suspended soil. After decanting C1, another 20 ml of water was added to the residual solid and the process repeated to obtain solution C_2_. The two solutions were combined and filtered through a 0.45 μm membrane filter. The concentration of stable chloride in this solution was determined by anion chromatography. From this concentration, and from the volume of water extracted by the distillation, the chloride concentration in the original groundwater could be deduced.

### Measurement of ^36^Cl/Cl by accelerator mass spectrometry

As a precaution against possible contamination of the ion source of the accelerator in the event that a sample had a high ^36^Cl/Cl ratio, the Cl isotopes in the soil water were diluted with a standard Cl solution with a low isotopic ratio (^36^Cl/Cl < 10^−15^). The standard solution was prepared from rock salt. Dilution factors were generally 1:10 (sample: rock salt), but were increased when chloride concentrations were low. Two samples were diluted 1:100. The mixture of the soil water and the standard solution was then filtered through a 3 kD ultrafiltration membrane to remove any organic matter that was dissolved in the soil. The Cl was precipitated out as AgCl after adding AgNO_3_ and acidifying with nitric acid. Sulphate was removed by dissolving the AgCl in NH_4_OH and adding Ba (NO_3_)_2_ to precipitate BaSO_4_ according to the procedure of Conard et al.^21^. AgCl was then reprecipitated from the supernatant by acidification with nitric acid. Approximately 20–50 mg of AgCl was obtained. The ^36^Cl/Cl ratios in the AgCl were measured by accelerator mass spectrometry at the Australian National University^22^.

### Measurement of tritium both by LSC and by the ^3^He ingrowth method using noble gas mass spectrometry

Soil waters were obtained by the distillation of the soil from the core for ^3^H analysis. Approximately 1000–2600 g of soil was placed in a stainless steel container connected to a glassware distillation system and heated to approximately 350 ℃ by a mantle heater. The distilled soil water was then electrolytically enriched in tritium by a factor of typically 30^23^. The enriched samples were mixed with an Ultima Gold uLLT™ scintillation cocktail and allowed to stabilize in the dark. The radioactivity of the tritium in the samples was measured by liquid scintillation counting using a Quantulus counter (PerkinElmer).

The concentrations of tritium from 6 samples (indicated by * in Table 1) were measured instead by the ^3^He ingrowth method^24^. Briefly, the distilled water sample was placed in a metal container with metal valves, and then all dissolved gases were completely extracted from it. The container was then sealed and stored for 48–52 days to allow ^3^He to grow in from the β decay of ^3^H. Following degassing of the sample, ^3^He was determined by noble gas spectrometry and the concentration of ^3^H in the soil water was deduced.

### Measurements of δD and δ^18^O

Additional soil water samples were separated for δD and δ^18^O analysis from sub-samples of the fresh undisturbed soil cores by centrifugation at 3500 rpm for 60 min. The water thus obtained was filtered through a 0.45 μm membrane filter and the δD and δ^18^O values were determined by stable isotope mass spectrometry^25^ (Supplementary Fig. S5). The δD and δ^18^O values show the soil water is originated from meteoric water.

### The one-dimensional migration analyses of ^3^H and ^36^Cl in the unsaturated soil column

A one-dimensional advective diffusion transport model^15^ was employed to predict the migration of ^3^H and ^36^Cl, both of which travel vertically with the water down the unsaturated soil column. The concentrations C(x,t) of ^3^H and ^36^Cl are functions of distance down the profile and time, and have the following form:

1 $$\frac{{C\left( {x,t} \right)}}{{ C_{0} }} = \frac{{Dx\exp (- \frac{{\left( {tv - x} \right)^{2} }}{4Dt} - \lambda t})}{{2\surd \pi \left( {Dt} \right)^{3/2} }}$$

where D is the dispersion coefficient and v is the vertical velocity. D was determined from the expression D = *α* × *υ* + *D*_*coff.,*_ where α is the longitudinal dispersivity, taken to be 0.02 m^26^, and D_coff:_ is the effective coefficient of molecular diffusion taken to be 0.02 m^2^ year^−1^^14^ in this study. The constant vertical velocity v of 0.95 m year^−1^ was deduced from the position (3.375 m) of the ^3^H peak. Equation (1) was solved with the initial condition that C(x,0) = 0, and an inlet boundary condition $-D\frac{\partial C\left(0,t\right)}{\partial x}+\upupsilon C\left(0,t\right)=\upupsilon \delta \left(t\right)$ corresponding to an instantaneous pulse input. The outlet boundary condition is $\frac{\partial C\left(\infty ,t\right)}{\partial x}=0$. The constant, C_0_ = C(0,t) = δ(t) = 1.0. A correction was made for radioactive decay of ^3^H. Vertical profiles of ^3^H and ^36^Cl deduced from this model for a range of times after the accident are shown in Figs. 2 and 3.

### Other

Weather data for Koriyama were obtained from the Japan Meteorological Agency. A topographic diagram map of the sampling sites (Fig. 1) was produced by GIS from the Geospatial Information Authority of Japan.

### Supplementary Information

Supplementary Information.

## Data Availability

All data generated or analyzed during this study are included in this published article and its supplementary information files.
