# Supplementary material for: Record of 3H and 36Cl from the Fukushima nuclear accident recovered from soil water in the unsaturated zone at Koriyama
Source: Sci Rep. 2023 Nov 11;13:19672. doi: 10.1038/s41598-023-46853-y (PMC10640602; doi:10.1038/s41598-023-46853-y)
Supplement: Supplementary file 1 — Supplementary Information. [file 41598_2023_46853_MOESM1_ESM.pdf]

Supplementary Information

Record of ^{3}H and ^{36}Cl from the Fukushima Nuclear Accident recovered from soil water in the unsaturated zone at Koriyama

Tomoko Ohta*[1,2], L. Keith Fifield[3], László Palcus[4], Stephen G. Tims[3], Stefan Pavetich[3]
, Yasunori Mahara[5]
[1]Nagaoka University of Technology, [2]The University of Tokyo, [3]Australian National University, [4]HUN-REN Institute for Nuclear Research, [5]Kyoto University

***corresponding author**
tomoohta@vos.nagaokaut.ac.jp

Supplementary Fig. 1. Map showing the relative positions and elevations of the 3 cores drilled at Koriyama in 2014, 2016 and 2019. The edited map is cited from the original map of (https://www.gsi.go.jp/tizu-kutyu.html) The map of the copyright holder is the Geospatial Information Authority of Japan.

Supplementary Fig. 2. Columnar section of sampling sites.

Supplementary Fig. 3. A cartoon showing how preferential flow through the old gophering hole near the 2014 drill hole could lead to elevated ^{3}H and ^{36}Cl in the groundwater below the water table at the base of the drill hole.

Supplementary Fig. 4. Observation of annual variation of the groundwater level at the Koriyama site.

Supplementary Fig. 5. δD (red square) and $\delta^{18}O$ (black square) as a function of depth in unsaturated soil water and shallow groundwater as measured from the soil core (6 m long) collected from Koriyama in 2014. The cross-hatched zone indicates the annual range of variability (~50 cm) in the groundwater table.

Supplementary Table 1. Observation of physical soil characteristics, ^{36}Cl and ^{3}H concentration and magnitude of depositional flux of ^{36}Cl and ^{3}H at Koriyama in 2014 and 2016 and at Yamakiya in 2016, in Fukushima Pref. Japan.

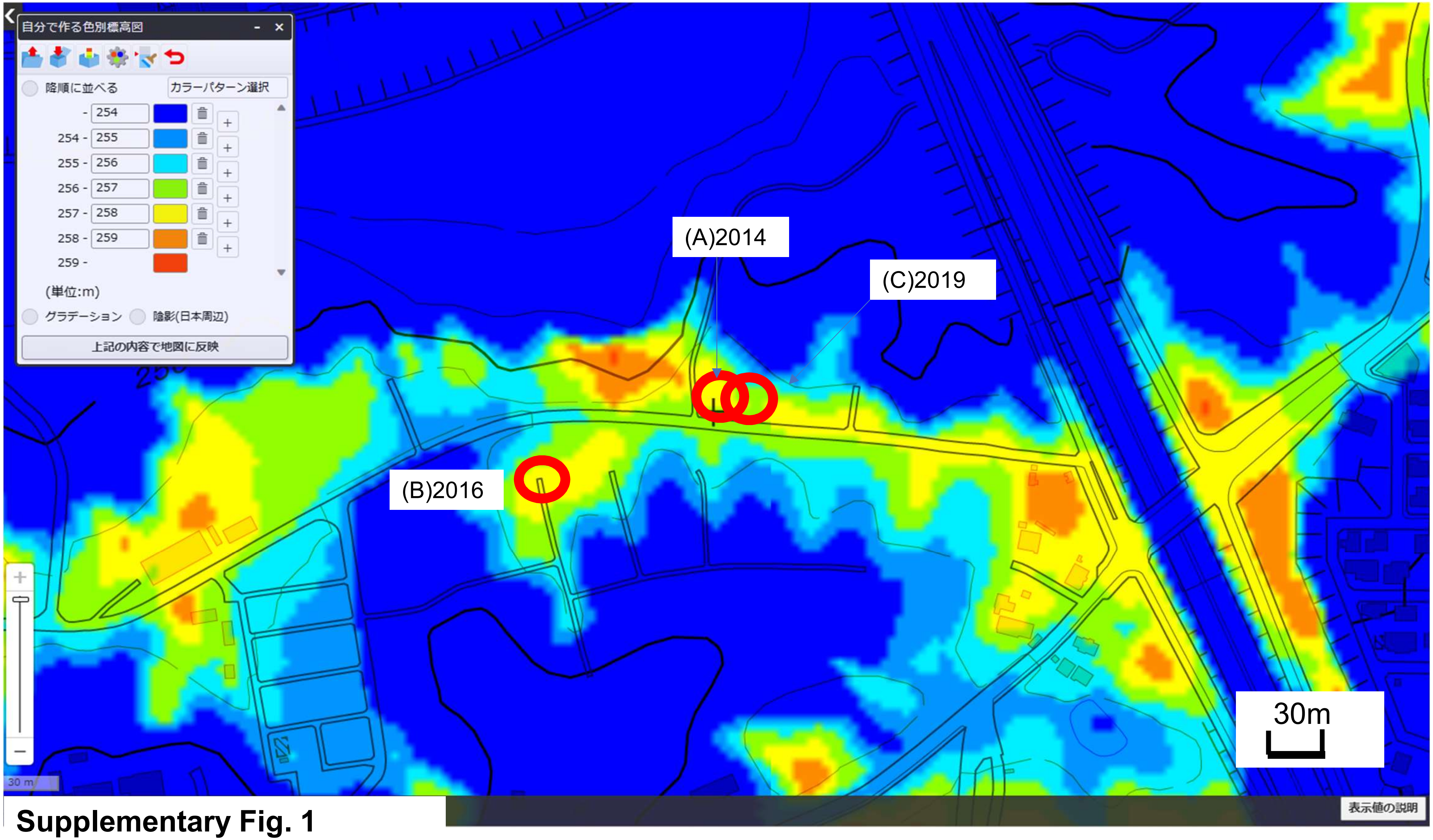

Supplementary Fig. 1

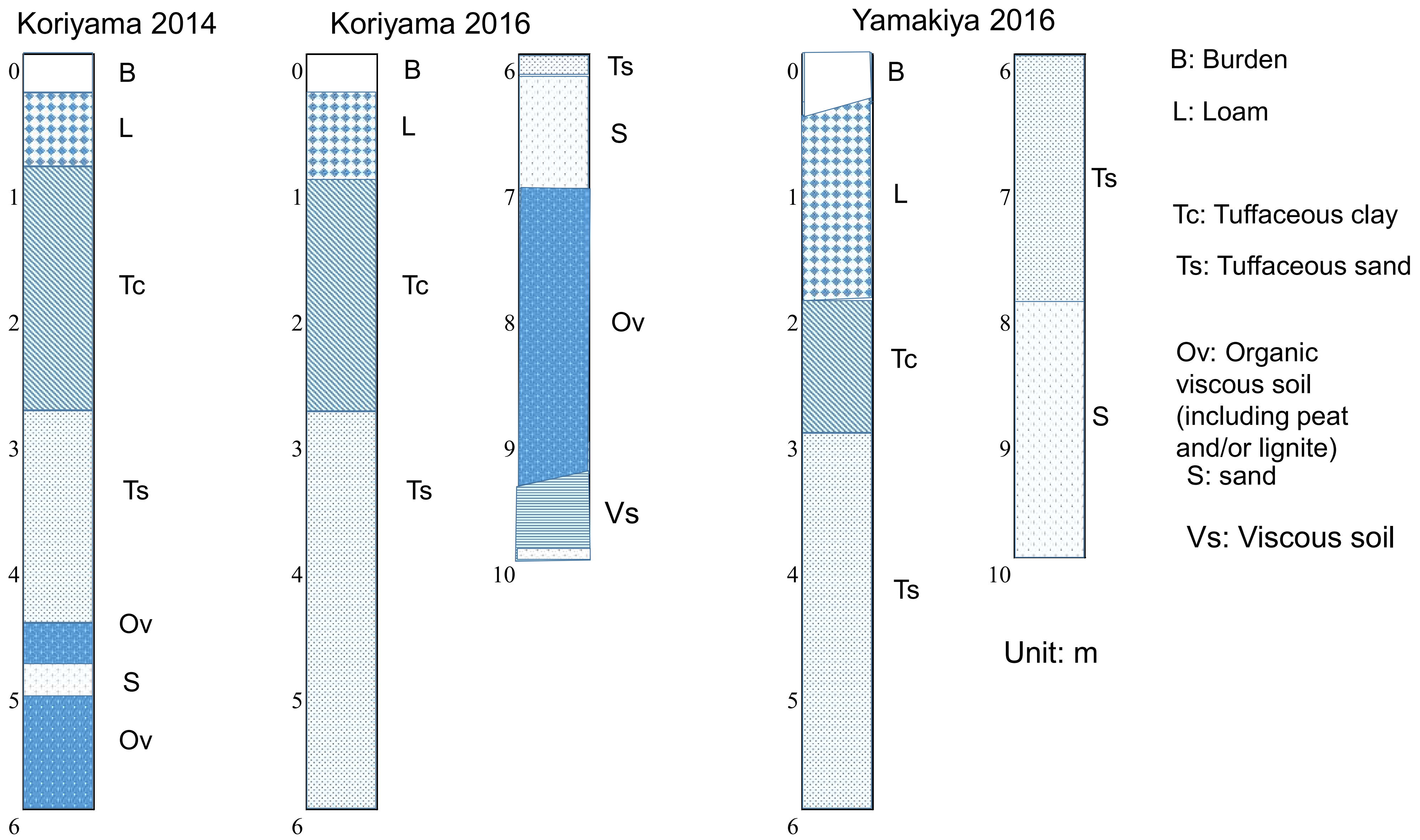

Supplementary Fig. 2

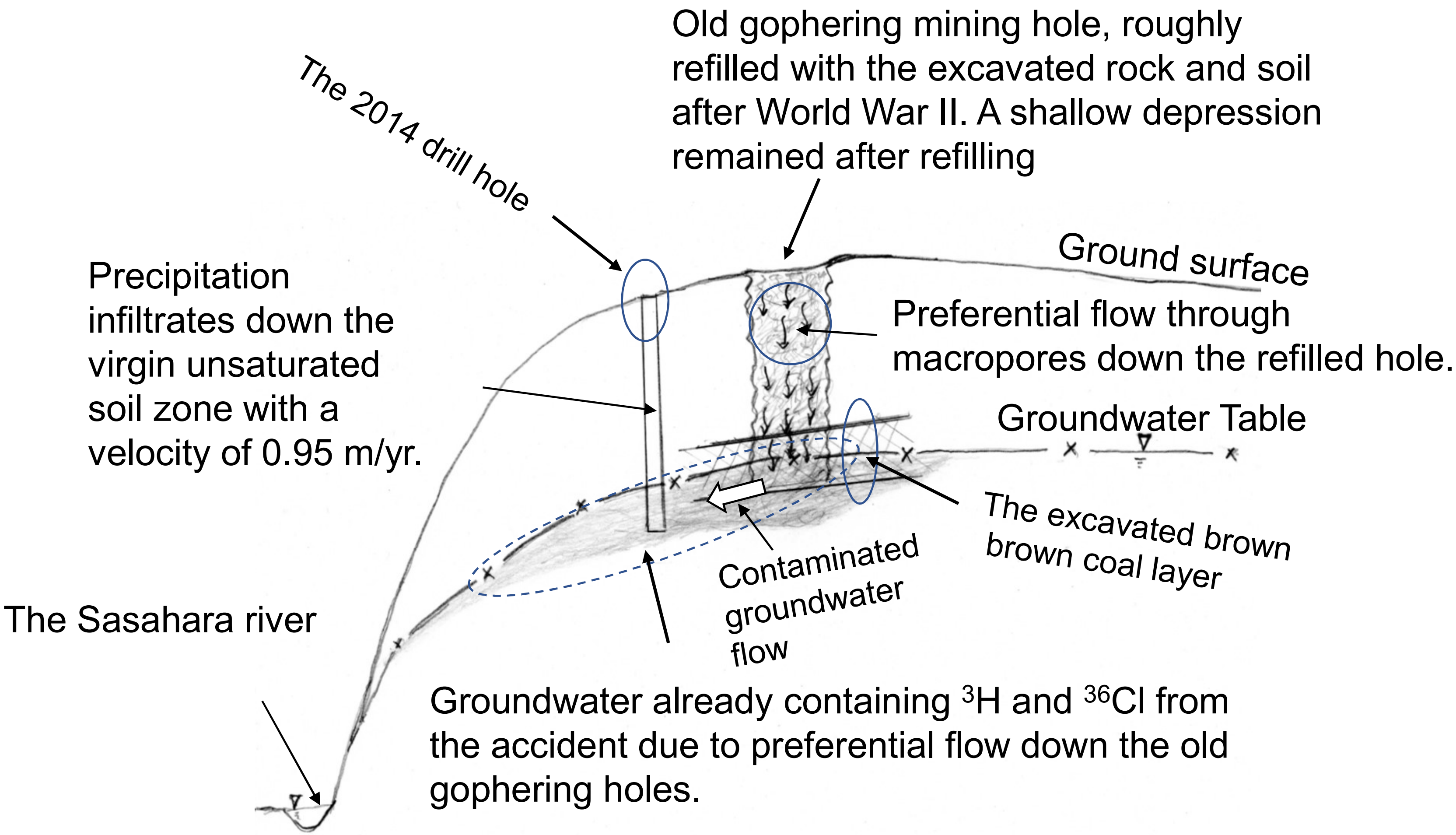

Supplementary Fig. 3

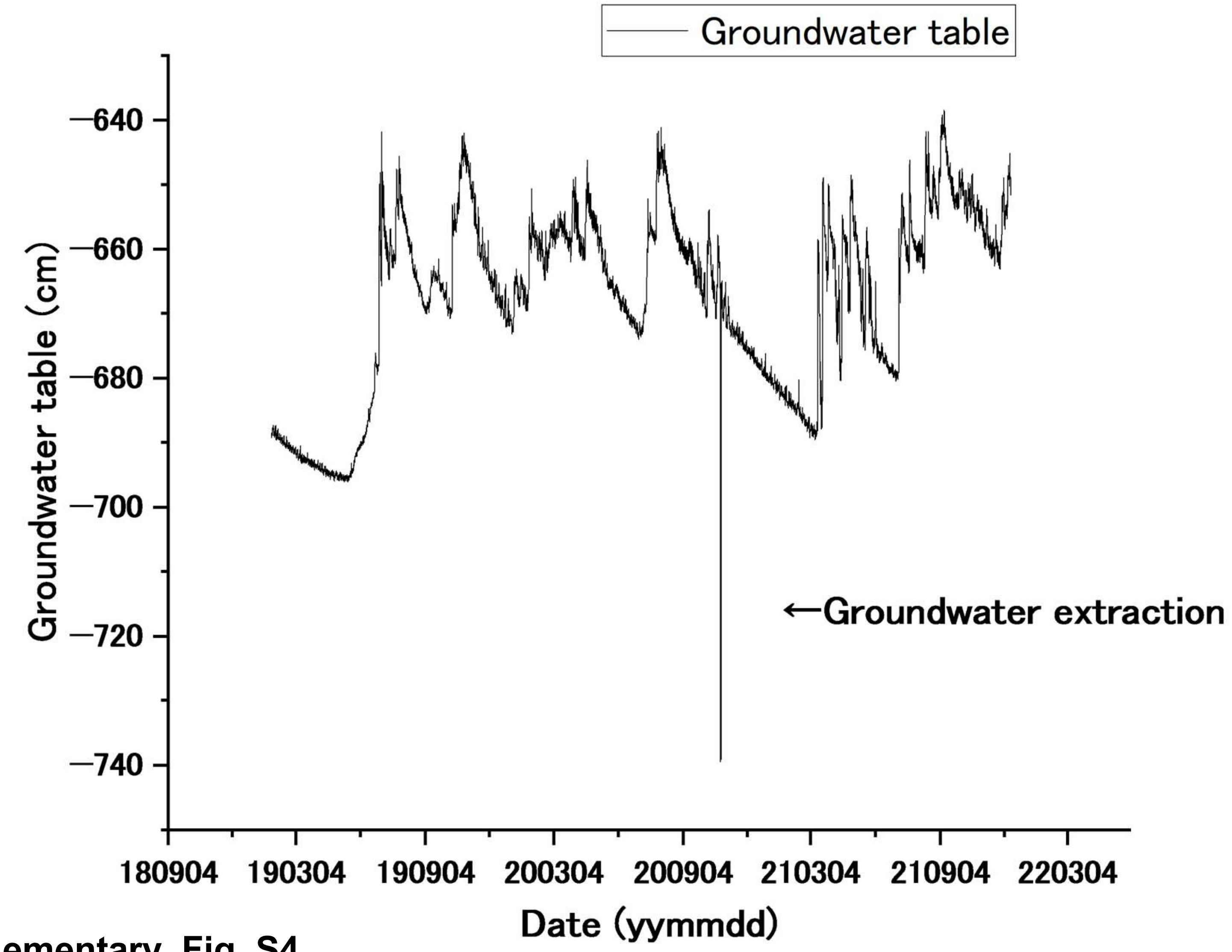

Supplementary Fig. S4

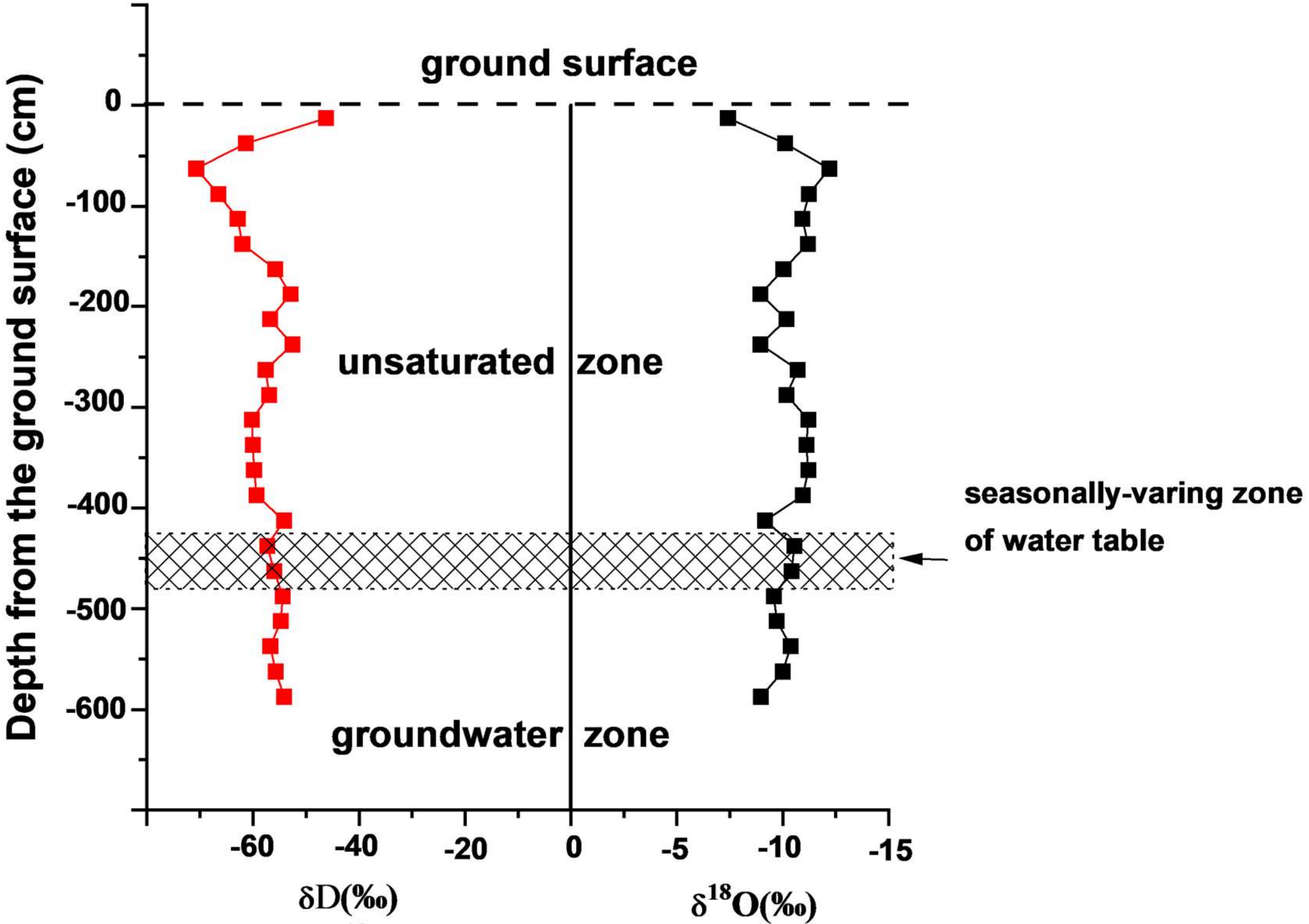

δD and δ^{18}O in the Koriyama 2014 core

Supplementary Fig. 5

Supplementary Table 1. Observation of Soil Physical Characterization, ^{36}Cl and ^{3}H Concentration and magnitude of depositional Flux of ^{36}Cl and ^{3}H at Koriyama (37.3576N, 140.3500E) in 2014 and 2016 and at Yamakiya (37.5920N, 140.7087E) in 2016, in Fukushima Pref. Japan.

Koriyama 2014 Depth (cm)		Core length (cm)	Sample ID	Moisture content (%)	Density of soil (g cm^{-3})	Porosity	Saturation	Volume of pore-water (cm^{3} cm^{-2})	Weight of soil (g cm^{-2})	^{3}H concentration (atoms L^{-1})	^{3}H Flux (atoms m^{-2}) (1Bq=5.61E8 atoms)	^{36}Cl (atoms L^{-1}) in soil water	^{36}Cl Flux (atoms m^{-2}) (1Bq=1.37E13 atoms)	^{36}Cl/^{3}H	^{3}H/6TU	^{36}Cl/natural**
-5	-25	20	KRFC1-1	27.9	2.255	0.562	0.679	7.632	19.75	6.02E+09	4.60E+11	2.01E+08	1.53E+10	0.03	15.06	43.66
-25	-50	25	KRFC1-2*	20.0	2.667	0.420	0.920	9.664	38.65	1.24E+10	1.20E+12	1.93E+09	1.87E+11	0.16	31.00	420.06
-50	-75	25	KRFC1-3*	19.5	2.630	0.440	0.812	8.932	36.82	6.30E+09	5.63E+11	8.63E+08	7.71E+10	0.14	15.76	187.53
-75	-100	25	KRFC1-4*	20.7	2.606	0.464	0.785	9.117	34.90	1.14E+10	1.04E+12	9.16E+08	8.35E+10	0.08	28.51	199.02
-100	-125	25	KRFC1-5*	24.6	2.624	0.505	0.839	10.60	32.45	5.00E+09	5.30E+11	1.15E+09	1.22E+11	0.23	12.49	250.47
-125	-150	25	KRFC1-6	24.0	2.641	0.477	0.916	10.92	34.52	3.55E+09	3.88E+11	4.61E+08	5.04E+10	0.13	8.88	100.31
-150	-175	25	KRFC1-7	30.8	2.684	0.572	0.892	12.76	28.72	7.24E+09	9.24E+11	9.58E+08	1.22E+11	0.13	18.11	208.30
-175	-200	25	KRFC1-8	27.7	2.667	0.497	1.033	12.42	33.56	6.81E+09	8.45E+11	1.23E+09	1.53E+11	0.18	17.02	267.25
-200	-225	25	KRFC1-9	30.1	2.575	0.564	0.855	12.07	28.04	6.76E+09	8.15E+11	1.74E+09	2.09E+11	0.26	16.89	377.18
-225	-250	25	KRFC1-10	28.5	2.598	0.543	0.874	11.86	29.71	9.00E+09	1.07E+12	9.50E+08	1.13E+11	0.11	22.50	206.63
-250	-275	25	KRFC1-11	26.7	2.617	0.499	0.957	11.92	32.81	9.07E+09	1.08E+12	1.27E+09	1.51E+11	0.14	22.67	275.20
-275	-300	25	KRFC1-12	20.5	2.562	0.486	0.697	8.468	32.93	9.81E+09	8.31E+11	3.03E+09	2.57E+11	0.31	24.53	659.19
-300	-325	25	KRFC1-13*	17.9	2.658	0.535	0.503	6.728	30.88	1.44E+10	9.70E+11	3.36E+09	2.26E+11	0.23	36.03	730.87
-325	-350	25	KRFC1-14*	15.6	2.677	0.537	0.429	5.749	31.01	2.58E+10	1.48E+12	2.33E+09	1.34E+11	0.09	64.54	506.47
-350	-375	25	KRFC1-15	27.9	2.650	0.546	0.852	11.64	30.07	9.13E+09	1.06E+12	2.14E+08	2.49E+10	0.02	22.83	46.60
-375	-400	25	KRFC1-16	25.5	2.650	0.525	0.818	10.74	31.45	2.82E+09	3.03E+11	4.00E+08	4.30E+10	0.14	7.05	87.00
-400	-425	25	KRFC1-17	21.4	2.648	0.462	0.839	9.694	35.62	2.71E+09	2.62E+11	2.63E+08	2.55E+10	0.10	6.77	57.16
-425	-450	25	KRFC1-18	23.6	2.657	0.516	0.773	9.961	32.18	7.79E+09	7.76E+11	6.65E+08	6.62E+10	0.09	19.47	144.50
-450	-475	25	KRFC1-19	47.7	2.527	0.708	0.950	16.82	18.46	9.09E+09	1.53E+12	5.96E+08	1.00E+11	0.07	22.72	129.56
-475	-500	25	KRFC1-20	44.6	2.599	0.705	0.879	15.48	19.20	8.19E+09	1.27E+12	2.32E+08	3.59E+10	0.03	20.48	50.35
-500	-525	25	KRFC1-21	66.9	1.913	0.804	0.942	18.93	9.38	9.54E+09	1.81E+12	2.09E+08	3.96E+10	0.02	23.86	45.48
-525	-550	25	KRFC1-22	53.5	2.289	0.725	0.999	18.11	15.76	7.13E+09	1.29E+12	6.22E+08	1.13E+11	0.09	17.84	135.15
-550	-575	25	KRFC1-23	40.0	2.420	0.616	1.004	15.40	23.23	7.60E+09	1.17E+12	3.94E+08	6.07E+10	0.05	18.99	85.65
-575	-600	25	KRFC1-24	31.2	2.522	0.525	1.034	13.12	29.97	8.12E+09	1.07E+12	5.87E+08	7.70E+10	0.07	20.31	127.62
													Average	0.07	17.50	95.83

*: Tritium concentration in soil water was measured by the ingrowth method

**: 4.6×10^{6} atoms L^{-1}

depositional Flux: inventory (Bq m^{-2}), * ^{3}H flux and ^{36}Cl flux: inventories of ^{3}H and ^{36}Cl

Koriyama 2016 Depth (cm)		Core length (cm)	Sample ID	Moisture content (%)	Density of soil (g cm^{-3})	Porosity	Saturation	Volume of pore-water (cm^{3} cm^{-2})	Weight of soil (g cm^{-2})	^{3}H concentration (atoms L^{-1})	^{3}H Flux (atoms m^{-2}) (1Bq=5.61E8 atoms)	^{36}Cl (atoms L^{-1}) in soil water	^{36}Cl Flux (atoms m^{-2}) (1Bq=1.37E13 atoms)	^{36}Cl/^{3}H	^{3}H/6TU	^{36}Cl/natural**
-35	-50	15	KFRC35-50	24.3	2.611	0.591	0.581	5.153	16.03	1.13E+08	5.83E+09	2.92E+07	1.51E+09	0.26	0.28	6.35
-135	-150	15	KFRC135-150	37.0	2.682	0.652	0.841	8.228	14.00	4.18E+08	3.44E+10	6.57E+07	5.41E+09	0.16	1.04	14.29
-273	-300	27	KY273-300	20.6	2.627	0.690	0.306	5.699	21.95	5.01E+08	2.86E+10	3.34E+07	1.90E+09	0.07	1.25	7.27
-392	-400	8	KY392-400	28.7	2.600	0.265	2.907	2.122	15.28	2.56E+08	5.44E+09	2.00E+08	4.25E+09	0.78	0.64	43.51
-480	-500	20	KY480-500	19.7	2.750	0.590	0.469	5.530	22.54	5.63E+07	3.11E+09	6.36E+07	3.52E+09	1.13	0.14	13.83
-530	-547	17.1	KY530-550	20.1	2.563	0.483	0.688	5.685	22.65	3.69E+08	2.10E+10	3.72E+07	2.12E+09	0.10	0.92	8.09
-581	-597	15.5	KY581-600	24.6	2.570	0.572	0.628	5.558	17.01	4.81E+08	2.67E+10	3.08E+07	1.71E+09	0.06	1.20	6.70
-635	-650	15	KFRC635-650	24.8	2.541	0.578	0.613	5.316	16.08	8.61E+07	4.57E+09	3.71E+07	1.97E+09	0.43	0.22	8.07
-732	-752	20	KFRC732-752	63.1	0.816	0.650	0.753	9.789	5.71	8.14E+07	7.97E+09	3.16E+07	3.10E+09	0.39	0.20	6.88
													Average	0.38	0.66	12.78

Yamakiya 2016 Depth (cm)		Core length (cm)	Sample ID	Moisture content (%)	Density of soil (g cm^{-3})	Porosity	Saturation	Volume of pore-water (cm^{3} cm^{-2})	Weight of soil (g cm^{-2})	^{3}H concentration (atoms L^{-1})	^{3}H Flux (atoms m^{-2}) (1Bq=5.61E8 atoms)	^{36}Cl (atoms L^{-1}) in soil water	^{36}Cl Flux (atoms m^{-2}) (1Bq=1.37E13 atoms)	^{36}Cl/^{3}H	^{3}H/6TU	^{36}Cl/natural**
-80	-100	20	YM-2016-4	38.6	2.525	0.776	0.459	7.114	11.33	2.54E+08	1.80E+10	1.15E+08	8.15E+09	0.45	0.63	24.91
-177	-194	17	YM-2016-8	49.1	2.452	0.852	0.408	5.913	6.15	3.28E+08	1.94E+10	3.04E+07	1.80E+09	0.09	0.82	6.60
-278	-295	17	YM-2016-12	16.8	2.595	0.616	0.327	3.424	16.93	7.35E+07	2.52E+09	5.21E+07	1.78E+09	0.71	0.18	11.32
-382	-400	18	YM-2016-16	14.9	2.593	0.499	0.454	4.078	23.38	2.77E+08	1.13E+10	3.14E+07	1.28E+09	0.11	0.69	6.84
-450	-465	15	YM-2016-19	15.5	2.814	0.642	0.288	2.778	15.09	2.41E+08	6.70E+09	5.70E+07	1.58E+09	0.24	0.60	12.39
-500	-515	14.5	YM-2016-21	15.7	2.481	0.548	0.381	3.031	16.25	5.24E+08	1.59E+10	5.13E+07	1.55E+09	0.10	1.31	11.15
-550	-565	14.5	YM-2016-23	14.9	2.490	0.449	0.538	3.501	19.89	4.28E+08	1.50E+10	5.59E+07	1.96E+09	0.13	1.07	12.14
-600	-612	12	YM-2016-25	15.0	2.486	0.568	0.336	2.289	12.89	2.90E+08	6.65E+09	4.27E+07	9.77E+08	0.15	0.73	9.28
-650	-664	14	YM-2016-27	14.9	2.523	0.495	0.449	3.113	17.85	3.28E+07	1.02E+09	4.09E+07	1.27E+09	1.25	0.08	8.90
-783	-800	17	YM-2016-32	14.5	2.511	0.456	0.507	3.934	23.20	4.37E+08	1.72E+10	8.25E+07	3.25E+09	0.19	1.09	17.94
													Average	0.33	0.73	10.73
